# A High Temperature Drop-On-Demand Droplet Generator for Metallic Melts

**DOI:** 10.3390/mi10070477

**Published:** 2019-07-17

**Authors:** Saeedeh Imani Moqadam, Lutz Mädler, Nils Ellendt

**Affiliations:** 1 Faculty of Production Engineering, University of Bremen, Badgasteiner Straße 1, 28359 Bremen, Germany; 2 Leibniz Institute for Materials Engineering IWT, Badgasteiner Straße 3, 28359 Bremen, Germany

**Keywords:** drop-on-demand, melt, metal, high temperature, droplet generator, micro sphere

## Abstract

In this study we present the design and functionality of a pneumatic drop-on-demand droplet generator that produces metallic micro particles with a size range of 300 µm to 1350 µm at high temperatures of up to 1600 °C. Molten metal droplets were generated from an EN 1.3505 (AISI 52100) steel which solidified during a falling distance of 6.5 m. We analyzed the resulting particle size and morphology using static image analysis. Furthermore, the droplet formation mode was analyzed using high-speed recordings and the pressure oscillation was measured in the crucible. The system is meant to be reproducible in all aspects and therefore the in-situ measurements are set to control the droplet size and trajectory during the run. Additionally, the ex-situ measurements are done on the particles in order to characterize them in size and morphology aspects.

## 1. Introduction

The generation of uniform molten metal droplets has many applications in science, ranging from the investigation of fundamental droplet solidification and droplet impact to novel applications such as placing solder balls on chips in the electronics industry, electrohydrodynamic (EHD) systems for film production or droplet-based additive manufacturing techniques and drop-on-demand spray forming [1,2,3,4,5]. There are two types of processes to obtain uniform droplets: continuous jet droplet generation and drop-on-demand techniques. Continuous jet droplet generators operate in a Rayleigh jet breakup mode and are excited by a vibrating rod in the melt to achieve a breakup mode free of satellite droplets. Such systems produce a continuous chain of uniform droplets which is well-suited for building three-dimensional objects or soldering applications at high production rates. Since the excitation is introduced with a piezo system, the temperature level is commonly restricted to low-melting point materials such as tin or zinc alloys.

Drop-on-demand droplet generators are able to generate triggered single droplets by introducing a momentum into the melt which pushes out the exact droplet volume. This momentum can be generated mechanically or pneumatically. Chandra and Jivraj initially developed the pneumatic drop-on-demand process for molten metals, in which the generation of a pressure oscillation firstly pushes out the melt volume and subsequently tears it back to let the formed droplet detach [6]. This volume is highly dependent on the characteristics of the pressure wave, so that the droplet size can not only be adjusted by the orifice diameter [7,8], but also by process parameters such as the pressure pulse time or amplitude. This process offers high reliability for research and has been successfully used for the analysis of droplet impact, droplet splashing and additive manufacturing [9,10,11]. While the original setup was limited to tin, zinc and aluminum alloys [12,13,14,15,16], Zhong et al. have extended the working range to copper [17]. In the past, we have shown that the cooling process of individual particles is highly reproducible and leads to well-defined microstructures. Therefore, the generated spheres may also be used as micro samples in a high throughput method to characterize and develop advanced new materials by predicting their properties [18].

**Figure 1:**
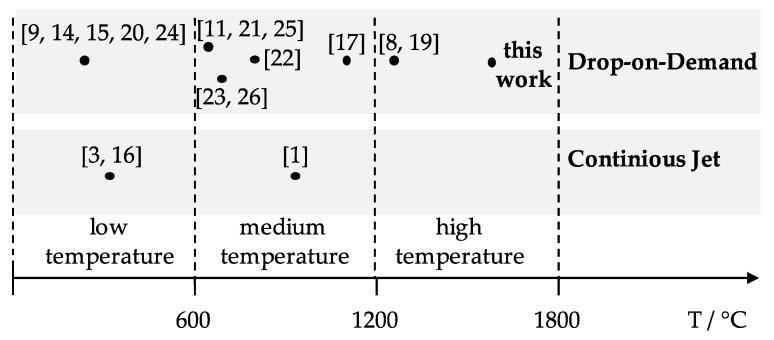
Overview of temperature ranges used in various droplet generation techniques [1,3,8,9,11,14,15,16,17,19,20,21,22,23,24,25,26].

Extending the process to higher temperatures suited for the processing of common steels and other materials in a similar temperature range requires major process developments such as the implementation of an inductive heating and a ceramic crucible/nozzle system resistant to such reactive melts. In this work, we present a pneumatic drop-on-demand setup which is capable of producing molten metal droplets at temperatures of up to 1600 °C. It features an alumina crucible/nozzle system and a falling distance of up to 6.5 m with modular quenching units at various heights. As illustrated in Figure 1, the range of the temperatures for various droplet generation techniques are different and, in this study, we are introducing a high temperature droplet generator that can be operated at higher temperatures than the previously introduced methods. In this study, exemplary experiments were carried out with EN 1.3505 (AISI 52100) steel and analyzed using high-speed recordings, in-situ measurements of the pressure wave and ex-situ particle size and shape analysis.

## 2. Experiments

### 2.1. Experimental Setup

**Figure 2:**
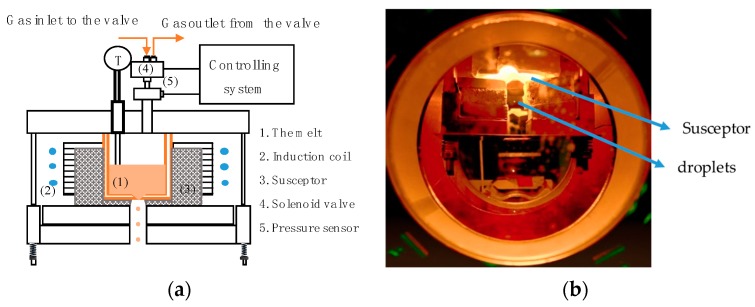
Experimental setup: (**a**) schematic view of the top plate of the droplet generator, (**b**) the view through the top window of the droplet generator during the experiment showing the glowing susceptor and the detached droplets during the process (Melt: EN 1.3505).

The device used was inspired by the design of Chandra et al. [6] and further designed to allow increased operating temperatures. The melting system (Figure 2a) consisted of an induction coil that heated a graphite susceptor. This susceptor conductively heated up the ceramic crucible and the contained metal to be melted. The indirect melting process can reduce the melt oscillations from the induction field to allow a stable droplet generation. Furthermore, it also allows low melt levels to be held at high temperatures, otherwise limited by direct inductive coupling. Figure 2b shows the generation of steel droplets (EN 1.3505).

**Figure 3:**
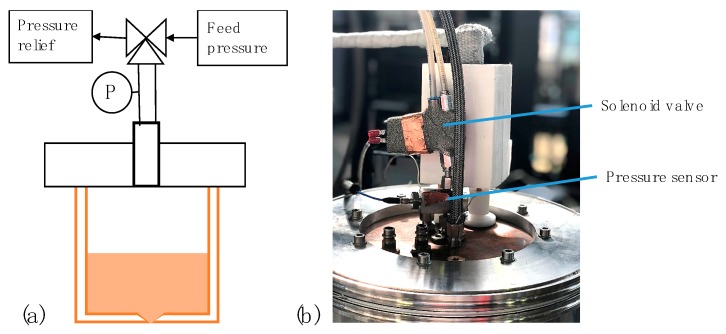
(**a**) Schematic view of the pressure sensor on the top plate of the droplet generator; (**b**) photo of the top plate of the droplet generator with removed cooling tubes.

The whole setup was placed in an inert chamber purged with nitrogen, argon or helium. To generate a droplet, the valve, which was subjected to a feed pressure, was opened for a short time (1–6 ms) and then switched back to open the crucible to the ambient atmosphere. This way, the pressure pulse introduced a pressure oscillation quickly released after the droplet had detached. The pressure wave in our droplet generator was measured via a dynamic piezoelectric pressure sensor (Model 112A21, PCB Electronics, Depew, NY, USA) with a sensitivity of 7.25 mV/kPa. The sensor was connected to a signal conditioner (Model 480E09, PCB Electronics, Depew, NY, USA) and recorded with an oscilloscope (Rigol DS1104). The pressure was measured at a point between the solenoid valve and the crucible. Figure 3 illustrates the location in the plant where the pressure sensor was located and where the dynamic pressure that enters the crucible was measured.

**Figure 4:**
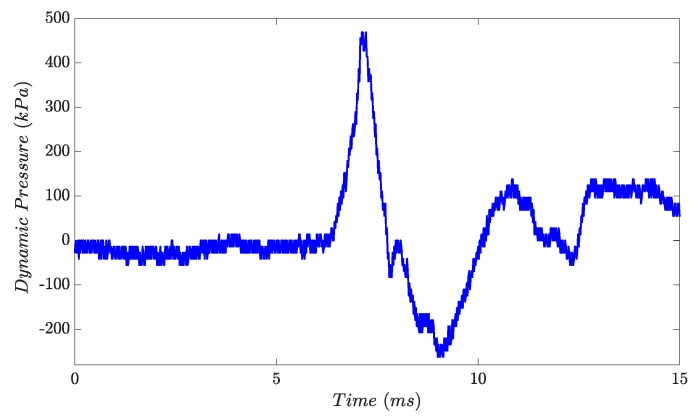
Pressure curve example for a valve opening time of 3.5 ms and a pressure of 69 mbar during an experiment with Copper (T = 1150 °C).

Figure 4 shows a typical pressure curve that can be obtained for copper or lower melting point metals. Each pressure pulse generates one droplet; more specifically, the pressure pulse lets the droplet form at the nozzle and detach afterwards with the pressure drop. During the experiment the pressure pulses were generated at a frequency of 10 Hz.

The particle size and velocity are related primarily by the droplet formation and separation process; this will differ depending on whether the droplet separation is controlled by underpressure or inertia. It also strongly depends on wetting properties which can be critical for metallic melts [27]. The variation of applied pressure also plays an important role.

**Figure 5:**
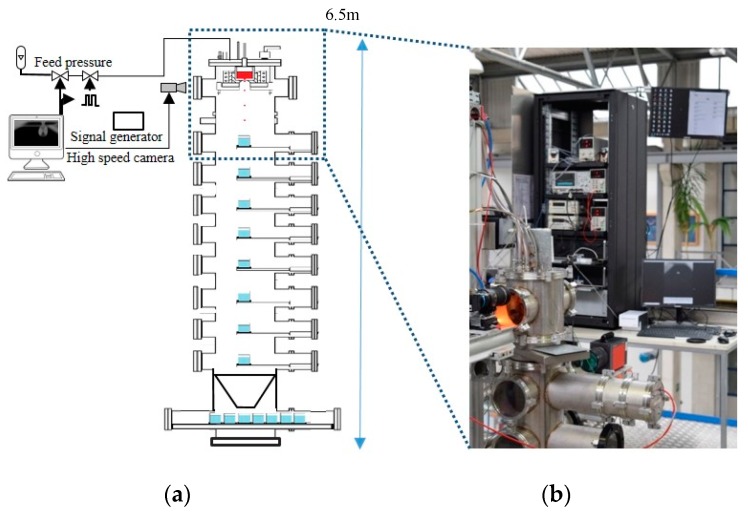
(**a**) Schematic view of high temperature drop-on-demand droplet generator; (**b**) the top part of the tower during the experiment showing the three top windows of the plant, high-speed camera and controlling systems.

The droplet generator head was mounted on a tower with a height of 6.5 m to provide enough falling distance for the droplets to solidify before they were collected. As displayed in the schematic view (Figure 5a), the glass collectors were either filled with oil or water, based on the melting material and corresponding temperature, and were installed in different levels in the tower to facilitate the collection of spheres in different phases during the solidification. Additionally, at the bottom of the tower, several collectors were set on a motor that could be moved during the experiment and therefore the resulting particles with known process parameters were amassed in each glass. A conic section was integrated in order to make sure that all particles were collected. (Figure 5a).

A high-speed camera ‘i-speed 210’ (Vizaar Industrial Imaging, Albstadt, Germany), was used to record droplet formation and initial droplet trajectories (Figure 5b). A scale of 69.15 pixels corresponding to 1 mm was used to allow quantitative analysis of the recorded images.

### 2.2. Experimental Parameters

Commercial EN 1.3505 steel was melted in an aluminum crucible to a temperature of 1560 °C in a nitrogen atmosphere. Once the material was fully melted, droplet generation was started by selecting a valve opening time and adjusting the feed pressure to find the stable droplet formation condition. The stability of the process was carefully checked with high-speed recordings. After stability was reached, the particle collector was replaced. The collectors were filled with Durixol WX61 quenching oil to cool down particles rapidly and avoid welding between particles. The main process parameters of the two experiments conducted in this study are summarized in Table 1. A high-speed recording of droplet formation for a valve opening time of 4 ms can be found in the Supplementary Material Video S1.

## 3. Analyses

### 3.1. High-Speed Recording of Droplet Formation and Determination of Initial Velocity of the Droplets

In order to visualize the formation of the droplets, they were monitored using high-speed imaging. The recording was done at a frame rate of 5728 fps. The recordings helped determine the droplet formation mode and were analyzed to monitor how the droplets differ in speed and in deviant angle [28].

The image series of the high-speed videos were analyzed after the experiments using ‘ImageJ 1.25a’ software to determine the droplet trajectory. For each setting, 3500 images were analyzed. Using this method, we could also calculate the initial velocity of the particles. The image analysis showed the reproducibility of the droplet generation and the process mode.

### 3.2. Static Image Analysis of Generated Particles

**Figure 6:**
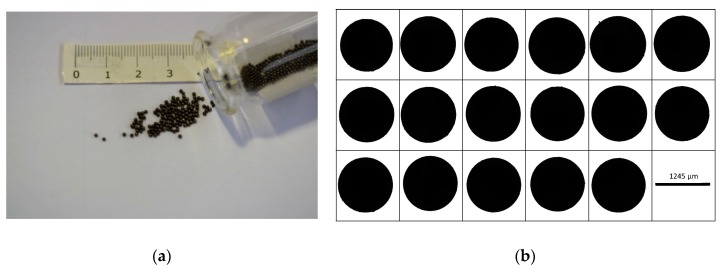
(**a**) Generated 100Cr6 particles after being cleaned and dried. (**b**) Overview of 17 different particles under G3 device.

After the experiment, the particles were cleaned of quenching oil and dried in a furnace at 80 °C. Figure 6 shows the particles that were generated during the experiments.

The particles were analyzed with respect to their size and morphology by static image analysis using the Malvern Morphologi G3 device with a combined magnification of 2.5× and 5× lenses.

#### 3.2.1. Circle Equivalent Diameter

Particle size can be uniquely defined for spheres by their diameter (or radius). For particles that are not completely spherical, the derived diameters are determined by measuring a size-dependent property of the particle and relating it to a single linear dimension [29]. Circle equivalent diameter is the most widely used and determined by:

dsurf=(4πAparticle)12

with *A_particle_* being the projection area determined from image analysis.

#### 3.2.2. Circularity

While there are several ways to define circularity [30,31,32,33], here we have used the ratio of the image area to the area of the circle of diameter *F_max_* in order to define circularity [34], namely:

$$C=\sqrt{\frac{4A}{\pi F_{max}^{2}}}=\sqrt{\frac{4A}{\pi L^{2}}}$$

The circularity is measured for each individual particle. The values are below 1 for non-circular projection areas and 1 for exact circles.

## 4. Results and Discussion

### 4.1. Determination of Pressure Wave

**Figure 7:**
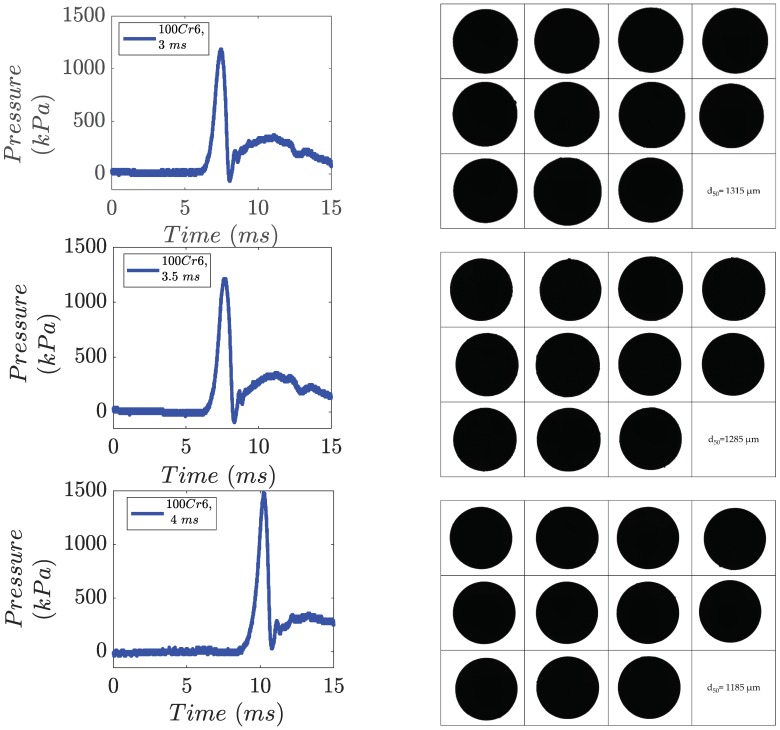
Pressure wave measurements during the experiments with EN 1.3505 and valve opening times of 3, 3.5 and 4 ms and corresponding particle images from image analysis.

The pressure behavior was analyzed and recorded during the experiments. Figure 7 shows the pressure wave recorded in two different experiments. During the steel experiments, the gas expands strongly in the crucible which leads to a loss of the under-pressure part of the curve obtained for lower melt temperatures.

### 4.2. Droplet Formation

**Figure 8:**
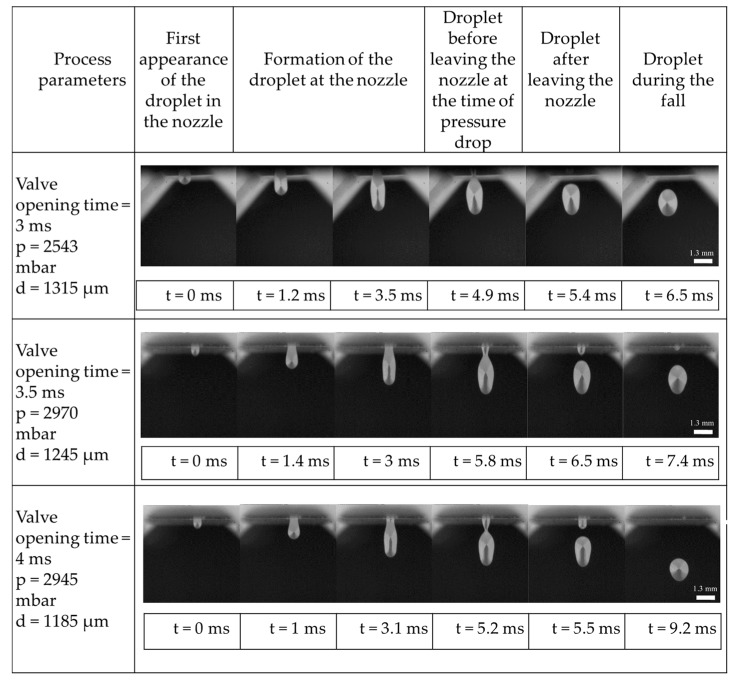
Droplet formation modes for different valve opening times and corresponding feed pressures p.

Figure 8 illustrates the formation and detachment process of the EN 1.3505 droplets during the experiment, in various modes. For each mode, the first appearance of a single droplet is taken as the starting point. By opening the valve and releasing the gas inside the crucible with the pressure pulse, the melt is let out and the droplet forms. Closing the valve results in a pressure drop which in turn lets the formed droplet leave the melt at the nozzle. The higher pressure, together with the valve opening time influence the size and velocity of the droplets. As seen in Figure 8, droplets that exit with smaller pressures are slower when leaving the nozzle. Finally, the droplet falls in the tower and is collected at a specified distance.

A jetting mode was present for all process conditions, in which a large ligament is pushed out and then after droplet separation, is torn back into the crucible. This is due to the lack of an underpressure section of the pressure curve; in this case, the melt is not actively torn back by an underpressure. Hence, it is the droplet inertia which controls droplet separation.

#### Image Analysis of High-Speed Recordings

**Figure 9:**
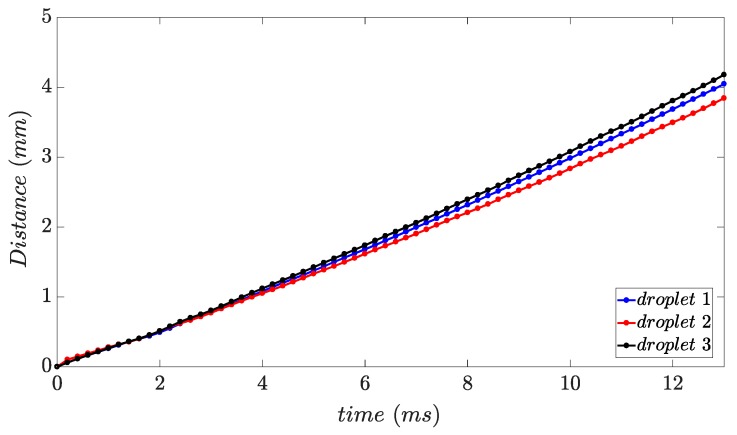
Droplet trajectories determined from high-speed recordings for three consecutive EN 1.3505 droplets during fall; valve opening time: 4 ms, 2945 mbar.

The reproducibility of the modes is illustrated by particle trajectories. Figure 9 shows the particle trajectories for three subsequent droplets during the same process mode. The image analysis shows that the distance and direction from which the droplets pass through are the same. Although the correspondence is, to a great extent, the same for various droplets of a mode at the beginning of the path, it is also seen that some droplets deviate from the trend as they get further from the nozzle. This could be caused by droplet size deviations resulting in different initial velocities of the droplets. The droplets could have been differently separated from the melt due to size variations, and therefore they would have different initial kinetic energies. The average initial velocity of the three droplets in Figure 9 is 0.267 ± 0.011 ms^−1^.

### 4.3. Particle Size Distribution and Circularity

**Figure 10:**
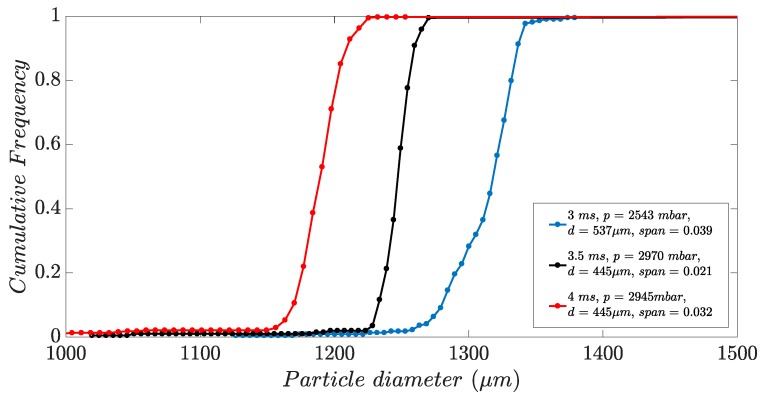
The cumulative sum for 100Cr6 particles in different process modes.

The distribution of the droplet size is narrow in the process of single droplet generation. The aim was to define the modes at which the particle size distribution is as narrow as possible. Figure 10 illustrates the cumulative sum of the particles generated during the experiment with 100Cr6 in three different process modes; where relative span is calculated as $\frac{d_{90}-d_{10}}{d_{50}}$ and ‘*d*’ is the diameter of the nozzle. We have observed the effect of various valve opening times on droplet size and as can be interpreted from the diagram, the smaller valve opening time led to bigger droplet size. However, the size distribution of the droplets also depends on the applied pressure in the system and the corresponding pressure curve.

**Figure 11:**
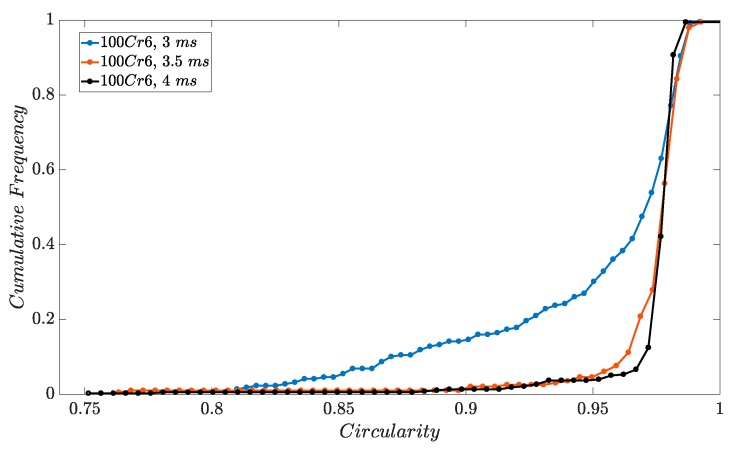
Average circularity of the spheres from image analysis, 0.973 ± 0.029.

Spherical particles indicate that solidification was complete at the time the particles were collected. Partially liquid particles would be deformed during impact. Hence, a high sphericity indicates that the solidification distance is long enough. For the largest droplets, the slight decrease of circularity shows that the generated particle size may be close to the solidification limit. To discuss the shape factors, the circularity of the produced spheres is taken into account. The results prove the hypothesis of generating spherical particles. Figure 11 presents the circularity of the particles determined from their projected images. With a mean standard deviation of 0.029 in the circularity of the particles’ projections for all three modes, the particles are almost completely spherical. The minimum sphericity comes from the larger droplets. This could be resulting from the fact that larger particles are not completely solidified as they enter the quenchant liquid and consequently, they deform as they collide with the bottom of the collector. Additionally, the particles may collide with the conical shape inside the tower if they do not fall precisely straight and this would result in a relative deformation in their spherical shape. The cone is designed to keep the particles from jumping to other glasses at the bottom of the tower (Figure 5a).

### 4.4. Process Window

**Figure 12:**
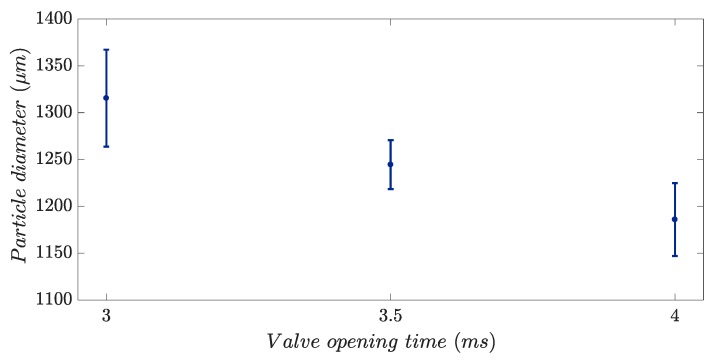
Process window for droplet size adjustment.

Finding a fitting pressure that would yield the generation of individual single droplets in the process is significant; double droplets or more are generated if the pressure is high and no droplets will come out of the nozzle if it is too low. The pressure, set manually during the experiment, was found for each valve opening time together to allow the stable droplet generation. Figure 12 shows the stable process conditions based on various valve opening times at which single droplets were obtained. The mean particle diameter (d_50,0_) is indicated with d_90,0_ and d_10,0_ as the error bars, over the valve opening time.

The particle size can be adjusted by changing the process parameters. It corresponds to the significance of a stable mode during the experiment. After several experiments with the same parameters and the same material, the parameters were set and defined as reproducible stable conditions for producing particles of certain characteristics. Figure 12 shows the process window for the experiments conducted in this study.

## 5. Conclusions

**Figure 13:**
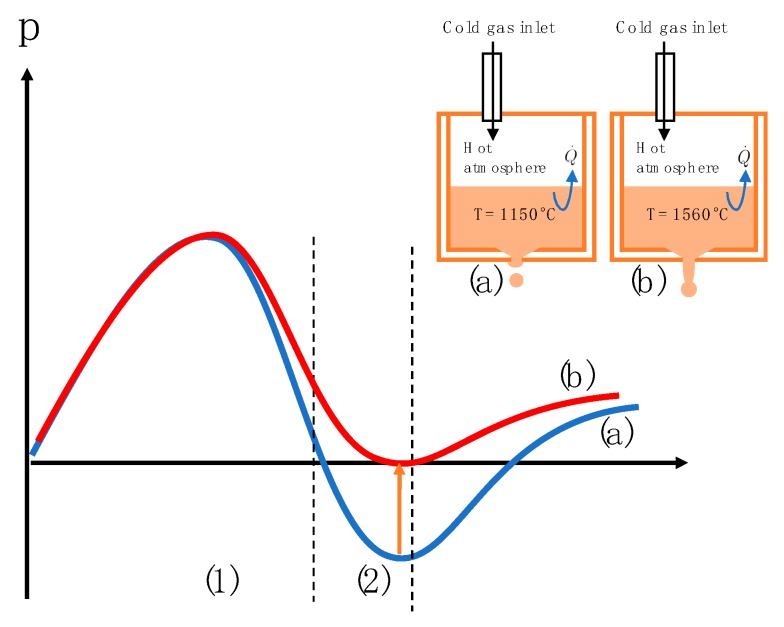
The pressure curve difference for medium melting metals (1150 °C), as in (a), and high melting metals (1560 °C), as in (b), as a result of gas expansion; interval (1) refers to the ejection phase of the droplet and interval (2) illustrates the separation phase.

In this work, a droplet generator was presented which extends the principle of pneumatic drop-on-demand molten metal droplet generation to high temperatures of up to 1600 °C with an all-ceramic crucible system. Experiments with EN 1.3505 steel at a melt temperature of 1560 °C have shown that reproducible drop generation is still possible at these process temperatures. Figure 13 shows the pressure curve difference for medium temperature (1150 °C) and high temperature (1560 °C) metals as a result of gas expansion. After the cold gas has entered the pressure chamber, it heats up in the crucible. For high temperatures, the gas expands strongly enough to prevent the formation of an underpressure range.

As illustrated, the droplet is ejected in the first phase and separated in the second phase. Since no underpressure range is present for high temperatures, the separation phase is controlled by inertia at high temperatures. As a result, only droplet formation in jetting mode could be achieved.

An evaluation of high-speed images showed that the droplet initial velocity is very reproducible, which indicates a high reproducibility of the droplet formation process. This is supported by the analysis of diameter and shape of the generated particles by a static image analysis. Finally, it could be shown that the droplet size can still be controlled by adjusting the pressure curve in the process. Possible applications include the use for additive manufacturing as already shown for lower melting point materials such as aluminum or the synthesis of material samples for high-throughput processes.

## Figures and Tables

**Table 1:** Experimental Parameters of the two experiments.

Alloy	100Cr6 (EN 1.3505)
Melt temperature (°C)	1560
Melt volume (cm^3^)	4.74
Crucible material	Al_2_O_3_
Nozzle diameter (µm)	537/445
Inert gas	N_2_
Type of quenchant	Quenching Oil Durixol WX61
Frequency (Hz)	10
Feed pressure (mbar)	2543, 2335, 2945
Valve opening time (ms)	3, 3.5, 4
distance (m)	6.581

## Supplementary Materials

The following are available online at https://www.mdpi.com/2072-666X/10/7/477/s1, Video S1: Droplet Formation, valve opening time: 4 ms.
